# Identification of QTLs conferring resistance to downy mildew in legacy cultivars of lettuce

**DOI:** 10.1038/srep02875

**Published:** 2013-10-07

**Authors:** Ivan Simko, Amy J. Atallah, Oswaldo E. Ochoa, Rudie Antonise, Carlos H. Galeano, Maria Jose Truco, Richard W. Michelmore

**Affiliations:** 1 U.S. Department of Agriculture, Agricultural Research Service, U.S. Agricultural Research Station, 1636 E. Alisal St, Salinas, CA 93905, USA; 2 The Genome Center and Department of Plant Sciences, University of California, Davis, CA 95616, USA; 3 KeyGene N.V., P.O. Box 216 6700 AE Wageningen, The Netherlands

## Abstract

Many cultivars of lettuce (*Lactuca sativa* L.), the most popular leafy vegetable, are susceptible to downy mildew disease caused by *Bremia lactucae*. Cultivars Iceberg and Grand Rapids that were released in the 18^th^ and 19^th^ centuries, respectively, have high levels of quantitative resistance to downy mildew. We developed a population of recombinant inbred lines (RILs) originating from a cross between these two legacy cultivars, constructed a linkage map, and identified two QTLs for resistance on linkage groups 2 (*qDM2.1*) and 5 (*qDM5.1*) that determined resistance under field conditions in California and the Netherlands. The same QTLs determined delayed sporulation at the seedling stage in laboratory experiments. Alleles conferring elevated resistance at both QTLs originate from cultivar Iceberg. An additional QTL on linkage group 9 (*qDM9.1*) was detected through simultaneous analysis of all experiments with mixed-model approach. Alleles for elevated resistance at this locus originate from cultivar Grand Rapids.

Cultivated lettuce (*Lactuca sativa* L.) is a diploid, autogamous species with 2*n* = 2*x* = 18 chromosomes. Lettuce cultivars are classified into horticultural types that differ morphologically. The most popular types produced for leaf consumption are iceberg with a large spherical head, romaine with an elongated head, butterhead with a small spherical head and pliable leaves with oily texture, and non-heading leaf-type lettuces with loose leaves. Analyses with molecular markers identified low genetic variability in iceberg and to lesser extent also in romaine and butterhead types^1^,^2^,^3^,^4^,^5^. Narrow genetic base may mean that these types are impoverished with respect to genes for resistance to pathogens relative to the lettuce genepools^6^. Consequently, lettuce breeders seek to exploit genetic diversity of wild progenitor species, different horticultural types, landraces, and old cultivars.

Downy mildew, caused by the biotrophic oomycete *Bremia lactucae* Regel, is one of the most economically important diseases of cultivated lettuce worldwide. Lettuce can be infected by this pathogen at any developmental stage, from young seedlings to mature plants. Infected plants develop yellow to pale green lesions that eventually become necrotic due to secondary pathogens following the breakdown of the biotrophic interaction. *B. lactucae* infection leads to lower marketable yield and to higher harvest-related expenses owing to the need to remove infected leaves. Chemical control of downy mildew with metalaxyl-based fungicides is possible, but ineffective against insensitive pathotypes^7^.

Two types of resistance to downy mildew have been described in cultivated lettuce. One is conferred by major, dominant single genes and a second that is polygenic in nature. Over thirty race-specific, single dominant genes or resistance factors (*Dm* or *R factors*) have been identified in lettuce^8^,^9^. Most of these genes confer high levels of resistance to the disease at all plant developmental stages. Many of the mapped *Dm* genes are located in three resistance-gene clusters on linkage groups (LGs) 1 (*Dm5/8*, *Dm10*, *Dm17*, *Dm43*, and *Dm45*), 2 (*Dm1*, *Dm2*, *Dm3*, *Dm6*, *Dm14*, *Dm15*, *Dm16*, and *Dm18*), and 4 (*Dm4*, *Dm7*, *Dm11*, *Dm44*, *Dm48*, and *Dm49*), with a single *Dm* gene, *Dm13*, located at LG 3^10^,^11^,^12^,^13^,^14^,^15^. Several of these resistance genes have been shown to encode nucleotide-binding site leucine-rich repeat (NB-LRR) resistance proteins^12^,^16^,^17^. Resistance based on single dominant genes, however, has not proved to be durable, because new races of the pathogen have evolved that render the race-specific resistance ineffective^18^,^19^.

Quantitative resistance has been documented in several lettuce cultivars^20^,^21^,^22^ that typically become infected with most races of the pathogen but the lesions are small and sporulation is limited. Often this phenotype is more evident in adult plants and hence its designation as adult plant, partial, or field resistance. In other pathosystems, some quantitative resistance has been shown to be determined polygenically and in certain cases has been more durable than single major genes^23^. Two of the most resistant lettuce cultivars with such resistance to downy mildew are Iceberg and Grand Rapids^24^ that were developed over a century ago and are not now used in modern lettuce production (note that cv. Iceberg is a Batavia rather than a modern iceberg type of lettuce, though both Batavia and iceberg are considered to be sub-types of crisphead lettuce). Cv. Grand Rapids was known in Michigan from at least 1880^25^. Cv. Iceberg was introduced to the U.S. from Europe in 1894, but its synonyms were mentioned in European catalogues as early as 1771^20^. Resistance to downy mildew in these cultivars appears to be durable as it was reported in Grand Rapids at least 50+ years ago^26^ and in Iceberg over 90 years ago^27^, although their lack of recent use has reduced their exposure to sustained selection for virulent races of the pathogen. Resistance in both cultivars is expressed only at young to mature plant stages; seedlings are readily infected by *B. lactucae*^28^,^29^. Previous genetic analyses of resistance using crosses that involved Grand Rapids and/or Iceberg revealed high estimates of narrow-sense heritability that suggested simple inheritance of resistance^21^. The segregation data, however, were consistent with multigenic modes of inheritance, such as two recessive genes, or two dominant genes with complementary gene action^21^,^22^,^30^. To investigate the genetic basis of resistance further, we developed a population of recombinant inbred lines (RILs) originating from a cross between cultivars Grand Rapids and Iceberg and analyzed this population for resistance to downy mildew in multiple field and laboratory experiments.

The objectives of the present study were to 1) develop genetic map of Grand Rapids × Iceberg (GR × Ice) population based on single nucleotide polymorphism (SNP) and amplified fragment length polymorphism (AFLP® markers, 2) align this map to the reference genetic map of lettuce^31^, 3) map quantitative trait loci (QTLs) for downy mildew resistance, and 4) evaluate stability of QTLs in different environments. The long-term objective is to introgress resistance QTLs from Grand Rapids and Iceberg into different horticultural types of lettuce using marker-assisted selection and to develop lettuce cultivars with potentially durable resistance to downy mildew.

## Results

### Molecular linkage map

**Figure 1:**
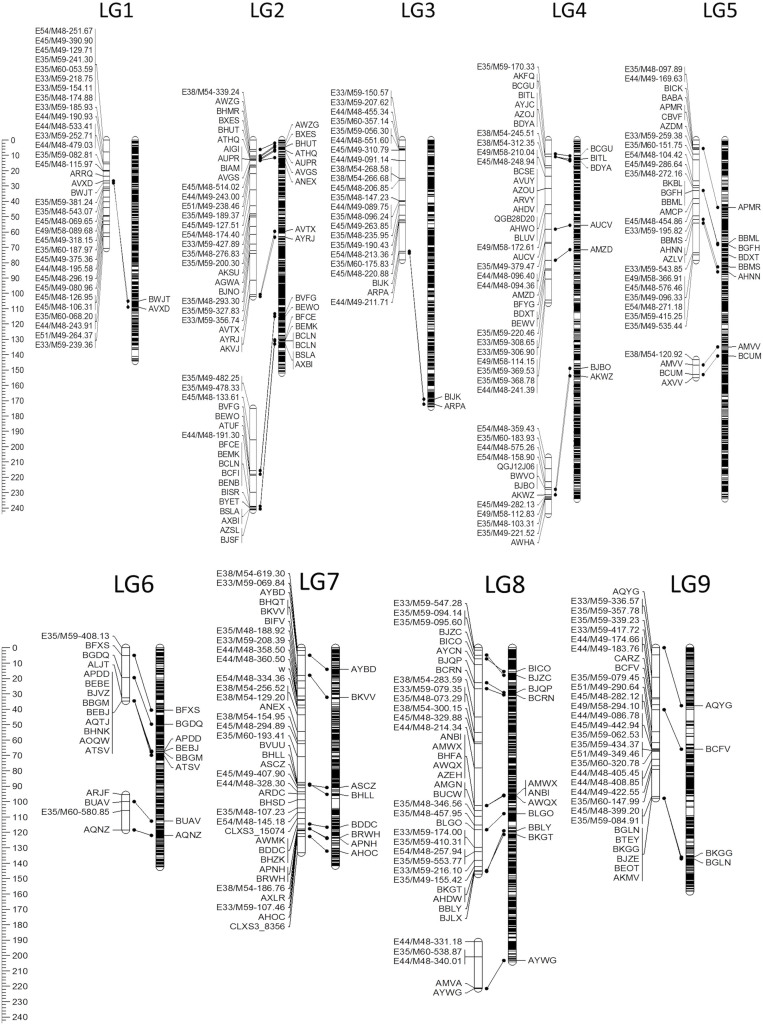
The Grand Rapids × Iceberg genetic linkage map. The map comprises of 131 SNPs, 176 AFLPs (names start with the letter E), and a single phenotypic trait for seed color (*w* gene) located on linkage group 7. Reference map of *L. sativa* x *L. serriola*^31^ is shown on the right in each linkage group. Scale in Kosambi centimorgans (cM) is on the left.

Genotyping of 90 RILs and parents with Illumina Golden Gate SNP assay and AFLP procedures produced 320 co-dominantly scored markers. Ten of the 141 SNPs (7.1%) and three of the 179 AFLPs (1.7%) did not group with other markers and were not used for linkage map construction. The GR × Ice linkage map thus comprised of 131 SNPs, 176 AFLPs, and a single phenotypic locus *w* for seed color. These 308 markers were mapped into 14 linkage groups with the total length of 1,017 centimorgans (cM). Eighty SNPs that were mapped both in the GR × Ice and reference linkage maps^31^ allowed assigning these 14 groups of markers to the nine chromosomal linkage groups of lettuce (Fig. 1). The length of the nine GR × Ice linkage groups (combined lengths of marker groups) ranged from 58 cM (LG 6) to 178 cM (LG 8) and the number of markers per linkage group ranged from 17 (LG 6) to 48 (LG 4). The GR × Ice map covered 64% of 1,585 cM of the reference linkage map (Table 1). Corresponding regions of the two linkage maps were concordant, with a possible exception of LG 2a and LG 8a where intervals between markers were ca. 1.5 × longer on the GR × Ice map (Fig. 1). Twenty-six mapped markers (11 SNPs and 15 AFLPs) showed significant deviation from the expected Mendelian ratio at *P* < 0.05. Most of these 26 markers were located on LG 9 (10 markers) and LG 4 (7 markers), indicating segregation distortion in these genomic regions. The segregation distortion was not related to the marker type, as both types of markers showed approximately the same frequency (8%) of markers that did not fit the expected ratio (11/131 SNPs; 15/176 AFLPs). The average distance between two successive markers on the GR × Ice linkage map was 3.3 cM, ranging from 2.1 cM at LG 1 to 4.7 cM at LG 8.

### Downy mildew disease

The five field experiments over four years relied on natural infections. Downy mildew appeared late in the F-2008 and F-2012a experiments allowing only a single disease assessment at harvest maturity (Table 2). Three disease assessments were made in experiments F-2009, F-2011, and F-2012b when *B. lactucae* infections started 8, 7, and 7 weeks after seeding, respectively. Susceptible check (cultivar Salinas) had disease ratings at harvest maturity of at least 4.5 on a 0 to 5 scale in all but one experiment (Table 2). In a single experiment (F-2012a), the mean rating of Salinas was 3.3, indicating lower disease pressure. The highest disease pressure was observed in F-2009 and F-2011 when the mean rating at harvest maturity for Salinas was 4.9 and 4.7 and for the RILs 2.4 and 2.1, respectively.

Laboratory experiments were scored twice at 7 and 12 days after inoculation (DAI) on a 0 to 3 scale. At 7 DAI, *B. lactucae* sporulated on 18% of RILs in L-2012a, and 30% of RILs and Grand Rapids in L-2012b. At 12 DAI, almost all RILs and both parents had disease scores of 2 or 3, indicating that no resistance genes in this population gave high levels of resistance at the seedling stage to the isolates tested. The profuse sporulation on most of the RILs 12 DAI precluded QTL analysis using data from this time point (Table 2).

In almost all field and laboratory experiments Iceberg showed higher resistance to downy mildew than Grand Rapids. However, both parents were always significantly (*P* < 0.01) more resistant than the susceptible checks with a single exception of Grand Rapids in L-2012b. In two experiments (F-2008 and L-2012a), the mean rating of the two parents was the same. Spearman correlations between disease scores assessed at harvest maturity in field experiments were significant (*P* < 0.01) and ranged from *r* = 0.29 between F-2009 and F-2012b to *r* = 0.55 between F-2012a and F-2012b, and between F-2008 and F-2012a (Supplementary Table S1). Correlations between field and laboratory experiments were variable and ranged from *r* = −0.01 between F-2008 and L-2012b, to *r* = 0.40 between F-2011 and L-2012a. All but one correlations for L-2012a experiment were higher (from *r* = 0.21 to *r* = 0.40) than were the correlations between L-2012b and field experiments (from *r* = −0.01 to *r* = 0.21). Correlation between the two laboratory experiments was not significant (*r* = 0.19, *P* = 0.073). Correlations for integrated data were generally high and ranged from 0.31 (with L-2012b) to 0.83 (with F-2012b). Pearson correlations had similar values (Supplementary Table S1).

Fifty-eight isolates of *B. lactucae* were collected from experiments in Salinas and surrounding fields from 2008 to 2012. Isolates were tested in laboratory on the set of differential cultivars expressing all known *Dm* genes to determine the (a)virulence phenotypes of the pathogen. Eighteen different virulence phenotypes were detected in isolates sampled from the field trials. The spectrum of isolates did not vary greatly from trial to trial. *Avr17* was detected in all isolates of the pathogen (Supplementary Fig. S2). This was consistent with field observations in unrelated experiments in the Salinas growing area with a differential set of cultivars; lettuce cultivars with *Dm*17 were disease free during 2008 to 2012. The other three most frequently detected avirulences were *Avr37* (in 85% of isolates), *Avr38* (in 81%), and *Avr36* (in 78%). In total, 14 different avirulences were detected in at least one isolate of the pathogen.

### QTL analysis

**Figure 2:**
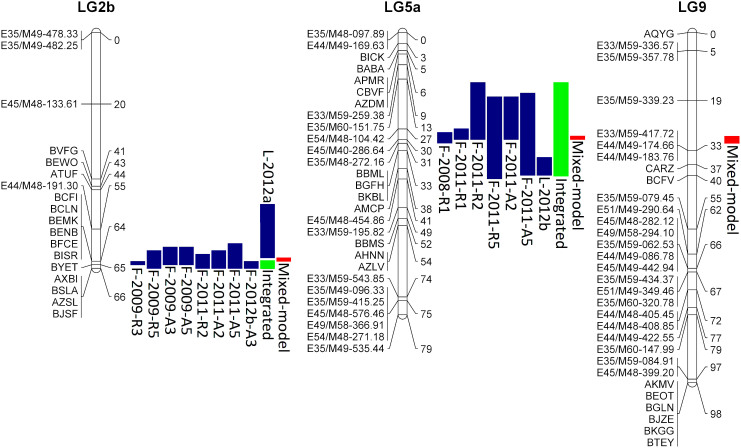
Location of *qDM2.1*, *qDM5.1*, and *qDM9.1* on the genetic linkage map of lettuce. Genetic distances in Kosambi centimorgans (cM) are indicated at the right of each linkage group. Blue and green vertical bars correspond to location of QTLs and 1-LOD support intervals calculated from individual experiments and integrated rating, respectively. Red bars show results from mixed-model analysis and support intervals calculated by QTLNetwork software. QTL *qDM2.1* is located on LG 2b, QTL *qDM5.1* is located on LG 5a, and *qDM9.1* is located on LG 9.

Five out of 20 datasets (F-2011 AUDPS, area under the disease progress steps) in week 2, F-2012a, F-2012b rating in week 1, L-2012a, and L-2012b) showed non-normal distribution at P < 0.05. These sets were therefore analyzed with both parametric and non-parametric tests. Because both tests identified the same QTLs, only results from parametric test are presented. Significant QTLs for resistance to downy mildew were detected in all experiments except F-2012a as well as in the integrated rating. The two QTLs were located on LG 2b (*qDM2.1*) and LG 5a (*qDM5.1*). QTL *qDM2.1* was detected in experiments F-2009, F-2011, F-2012b, L-2012a, and integrated rating (Table 3). Two SNP markers, BISR and BYET, were located within 1-LOD (logarithm of odds) support intervals of maximum LOD scores in nine data analyses (Fig. 2). These SNPs are positioned at 64.3 cM and 64.9 cM of LG 2b, respectively. The largest effect of *qDM2.1* was observed in F-2009 experiment, when LOD score of 4.8 and R^2^% (percent of the total phenotypic variance explained by the QTL) of 22% were recorded. RILs having *gg* (Grand Rapids) genotype at the BYET marker had the mean disease rating of 2.60, while those with *ii* genotype (Iceberg) had the mean disease rating of 1.64, indicating that the Iceberg alleles determined the elevated resistance. Disease ratings calculated at the BISR locus were almost identical, because only a single RIL has different genotypes at BYET and BISR loci.

QTL *qDM5.1* was significant in experiments F-2008, F-2011, L-2012b, and the integrated rating. This QTL is linked to AFLP marker E45/M49-286.64 located on LG 5a at positions 29.8 cM (Fig. 2). The largest LOD score of 4.6 was calculated for disease scores assessed at F-2011 experiment, when the QTL explained up to 21% of the trait variance. Similar to the QTL on LG 2b, the alleles of *qDM5.1* from cv. Iceberg determined elevated resistance (Table 3).

Neither of the QTLs mapped to the clusters of known *Dm* genes. However, alignment of the lettuce genome sequence and the ultra-high density linkage map revealed that *qDM5.1* is located close to a gene potentially encoding a TIR-NBS-LRR disease resistance protein for pathogen recognition. Two genes annotated as encoding disease resistance proteins (TM-LRR) were previously mapped to LG 2b^12^, but these sequences could not be accurately aligned to the GR × Ice map because of an insufficient number of common markers.

Analysis of resistance data with mixed-model composite interval mapping (MCIM) confirmed significant effects of *qDM2.1* and *qDM5.1*. In addition to these two QTLs, MCIM identified a third QTL (*qDM9.1*) on LG 9 (Table 4) near AFLP marker E33/M59-417.72. This QTL was not detected as significant when experiments were analyzed separately, with LOD scores ranging from 0.9 to 2.1. The additive effect of this QTL assessed on combined data was 0.088 what is ca. 60% to 75% of the additive effects of QTLs *qDM5.1* and *qDM2.1*, respectively. However, while alleles conferring elevated resistance at QTLs *qDM2.1* and *qDM5.1* originated from cultivar Iceberg, elevated resistance at *qDM9.1* was associated with Grand Rapids alleles. No significant QTL × QTL or QTL × environment interaction was detected for these three QTLs at P ≤ 0.001.

### Potential for marker-assisted selection

**Figure 3:**
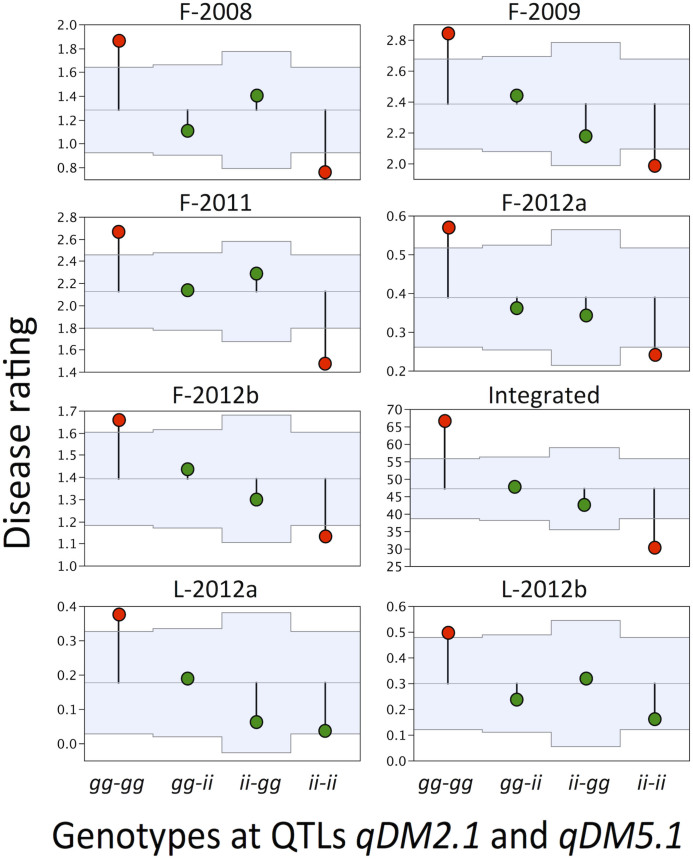
Testing potential of molecular markers for marker-assisted selection (MAS) with analysis of means (ANOM). The ANOM tests which of the four allelic combinations are associated with resistance that is significantly different from the overall resistance in the population. Areas in light blue show upper and lower bounds of decision limits. Values that are outside decision limits (marked in red) are significantly different (*P* < 0.05) from the overall mean. ANOM analyses were conducted on last disease ratings from field experiments, sporulation of pathogen 7 days after inoculation in laboratory experiments, and integrated data of these seven experiments. QTL genotypes are based on molecular markers BYET and E45/M49-286.64 that are closely linked to *qDM2.1* and *qDM5.1*, respectively. The first two letters of genotypes show alleles at *qDM2.1* and the other two letters show alleles at *qDM5.1*. Only recombinant inbred lines homozygous for either Grand Rapids alleles (*gg*) or Iceberg alleles (*ii*) were considered in the analysis.

A combination of two markers that were detected in multiple experiments, BYET at LG 2b and E45/M49-286.64 at LG 5a, was used for testing potential for marker-assisted selection (MAS) in this population. An analysis of field experiments and integrated data yielded similar results showing that the *gg*-*gg* combination of alleles had significantly (*P* < 0.05) higher disease rating than was observed for the population mean (Fig. 3). On the other hand, the *ii*-*ii* combination of alleles was always associated with significantly lower disease rating. In both laboratory experiments, only the *gg*-*gg* allelic combination was associated with significantly earlier sporulation, while the *ii-ii* allelic combination was not significantly different from population mean.

## Discussion

The GR × Ice linkage map covered approximately 64% of the reference genetic map, with coverage ranging from 38% for LG 5 to 111% for LG 2. The greater map length observed for LG 2 could have been the result of increased recombination in this intra-specific population or a variety of technical events such as using different software, different mapping parameters, or calculating the total length of this linkage group from two separate groups (LG 2a and LG 2b) of markers.

Segregation distortion observed on LG 9 (10 out of 31 markers), and LG 4 (7 out of 48 markers) was previously observed for these linkage groups in both intraspecific^32^ (Hayes and Simko, unpublished results) and interspecific^32^ lettuce crosses. Segregation distortion could have been caused by a number of factors including genetic factors^33^, or inadvertent selection during development of the RILs. The 8% frequency of markers with distorted segregation observed in the GR × Ice population is similar to the values of 6.32% and 7.78% that were reported for populations originating from other intraspecific lettuce crosses^32^.

The mean disease scores for the GR × Ice RILs tested in the five field experiments in Salinas ranged from 0.4 in F-2012a to 2.4 in F-2009. The low disease score recorded for RILs in the summer experiment (F-2012a) was congruent with the low rating of the susceptible cultivar Salinas (3.3 out of 5.0). These results are consistent with previous observations that disease pressure is usually higher in the Salinas area in fall growing seasons, due to prolonged morning leaf wetness and cool morning and midday temperatures^34^ that favor downy mildew development. Low disease pressure in F-2012a prevented accurate resistance scoring and QTL detection from this experiment. Similarly, no significant QTLs were detected in F-2009 in week one and F-2012b in weeks one and two when disease scores were still low. These results show that accurate assessment of quantitative resistance in this mapping population in the field requires high disease pressure. Detection of QTL may be improved by combining individual disease scores into AUDPS^35^, by joining data from individual experiments into an integrated rating^36^, or by using mixed-model analyses^37^.

Two significant QTLs for downy mildew resistance were detected in the present study when experiments were analyzed individually. The QTL *qDM2.1* was closely linked to the SNP markers BYET and BISR on LG 2b, while *qDM5.1* was linked to the AFLP marker E45/M49-286.64 on LG 5a. The *qDM2.1* was detected in F-2009, F-2012b, and L-2012a; *qDM5.1* was detected in F-2008, and L-2012b and both QTLs were detected in F-2011, and the integrated data. The two QTLs were effective both in Salinas, California and The Netherlands field experiments. Because isolates of *B. lactucae* collected in California and Europe differ in their avirulence phenotypes^38^,^39^, it appears that *qDM2.1* and *qDM5.1* provide resistance against races of the pathogen present at both locations. An additional QTL was detected on LG 9 only through simultaneous analysis of all experiments using a mixed-model approach. In contrast to QTLs *qDM2.1* and *qDM5.1*, the allele for elevated resistance at *qDM9.1* originates from cv. Grand Rapids. The additive effect of *qDM9.1* is 25% to 40% smaller than the additive effect of the other two QTLs. Because of its relative small effect and the fact that the QTL was not detected in any of the individual experiments, this QTL was not tested for its potential in MAS.

Testing of *qDM2.1* and *qDM5.1* potential for MAS revealed that combination of two markers closely linked to the resistance QTLs could accurately predict phenotypes in this population. When Iceberg alleles were present both at BYET and E45/M49-286.64 markers (*ii-ii*), resistance in field experiments and integrated data was significantly higher than population mean. In contrast, presence of Grand Rapids alleles at both markers (*gg-gg*) was associated with significantly lower resistance in all experiments. Selection based on the combination of BYET and E45/M49-286.64 markers would be useful even in the F-2012a experiment that did not yield any significant QTLs due to low disease pressure; RILs with *gg-gg* allelic combination had the mean disease score of 0.57 (95% confidence interval CI95: 0.40–0.74), while those with *ii-ii* alleles had the mean disease score of 0.25 (CI95: 0.17–0.33).

Over 20 *Dm* genes have been positioned on the lettuce linkage map^11^,^12^,^13^,^14^,^15^. These dominant genes clustered on LG 1 (five *Dm* genes), LG 2 (eight *Dm* genes), LG 4 (six *Dm* genes), and a single gene was detected on LG 3. None of *qDM2.1*, *qDM5.1*, or *qDM9.1* is located in these resistance gene clusters. The QTL on LG 2b is well separated from the *Dm* gene cluster on LG 2a. *Dm13* is present in both Grand Rapids and Iceberg^21^; however, *Dm13* was ruled out as contributing to the QTLs detected because all isolates of *B. lactucae* from Salinas in present and previous studies^21^,^40^ are not recognized by *Dm13*, the *Dm13* gene is located on LG 3 12 and therefore unlinked to *qDM2.1*, *qDM5.1*, and *qDM9.1*, and both parents are homozygous for *Dm13*, therefore *Dm13* does not segregate in this population.

Interestingly, *qDM2.1* resides within the same chromosomal region as genes for quantitative resistance to downy mildew *rbq5*^41^ and powdery mildew *pm-2.2*^35^. The *rbq5* gene originates from *L. saligna* and provides a partial resistance to plants at all developmental stages, including the seedling stage. However, resistance in young and adult plants harboring the *rbq5* gene is lower than in Iceberg plants at the same developmental stage^29^. Additional fine mapping of these QTLs is required to refine their genetic positions further and to facilitate their cloning. This will lead to the elucidation of the molecular basis of this quantitative resistance and indicate whether it has a fundamentally different molecular basis than that conferred by NB-LRR proteins and may therefore be more durable^23^.

Quantitative resistance in cultivars Iceberg and Grand Rapids (also called field resistance or partial resistance) was previously observed at the mature plant stage, while seedlings were susceptible to *B. lactucae* infection^28^,^29^. In our laboratory experiments, most of the RILs and both parents were heavily infected at 12 DAI, consistent with earlier studies. However, when linkage analysis was performed on data collected at 7 DAI, detected QTLs coincided with those found in adult plants tested in field conditions. These results indicate that *qDM2.1* and *qDM5.1* are active at the seedling stage; however, the QTLs do not prevent infection of seedlings by the pathogen, but rather only delay sporulation. Longer latent period to sporulation was reported for Iceberg seedlings when this cultivar was compared to other cultivars with varying levels of field resistance^28^.

Grube and Ochoa^21^ analyzed *F_2_*-derived populations from crosses that included Grand Rapids and Iceberg. Results of that study indicated that resistance in the GR × Ice population is based on two recessive genes, or two dominant genes with complementary gene action. The present study is consistent with the previous hypotheses of a low number of resistance genes and the presence of least one allele for resistance in each cultivar. Additional genes for quantitative resistance, however, may exist in these cultivars, but were not detected due to the limited population size that could be evaluated, coverage of the linkage map, or because both parents are homozygous for resistance gene(s).

A single RIL (GRxI-1053) consistently showed ratings lower than Iceberg in all field experiments. This RIL was highly resistant, with mean values of 0, 0.2, 0.2, 0.2, and 1, while Iceberg values ranged from 0.4 to 1.5, and Grand Rapids from 0.6 to 2. Integrated rating on a 0 to 100 scale was equal to 0 for GRxI-1053, 38.1 for Iceberg, and 65.6 for Grand Rapids. Detection of additional transgressive segregants with field resistance better than that of Iceberg was obstructed by the low disease rating for Iceberg in most of the experiments. Eight RILs had disease ratings higher than Grand Rapids in all field experiments. Six of these RILs have Grand Rapids (*gg*) alleles both at *qDM2.1* and *qDM5.1*, and Iceberg alleles (*ii*) at *qDM9.1*.

The GR × Ice population was used to detect resistance alleles originating from Iceberg, a 240+ years old cultivar according to available records. This 18^th^ century lettuce possesses one of the highest, possibly the highest level of quantitative resistance to downy mildew observed to date. Extensive testing of this legacy cultivar in numerous field trials over many years^20^,^21^,^22^,^24^,^27^,^29^,^42^ indicated that Iceberg alleles consistently provide quantitative resistance to downy mildew in young and adult plant stages. The resistance loci from Iceberg, however, have not been introgressed into modern cultivars, due to difficulties with accurate phenotyping and insufficient knowledge about their inheritance. Results of the present study will be used to develop molecular markers that can aid future marker-assisted selection programs, thus complementing field-based phenotyping. We are developing additional mapping populations from crosses with highly susceptible accessions that will be used to map resistance genes not detected in the present study. Seeds of GR × Ice RILs with the highest resistance to downy mildew are available for distribution upon request to the first author.

## Methods

### Development of mapping population and linkage map

Mapping population consisted of 90 *F_6_* RILs that were derived from a Grand Rapids × Iceberg cross using single seed descent. Grand Rapids is a non-heading leaf type of lettuce with black seeds. Iceberg is a Batavia type of lettuce that forms a loose head and has white seeds. DNA was extracted from five randomly selected plants of each RIL and the parents that were pooled into a single sample for each genotype. Tissue for DNA extraction was obtained from young leaves of about one month old plants and immediately lyophilized. Lyophilized samples were ground to fine powder using TissueLyser mill, before extracting genomic DNA with NucleoSpin Plant II kit (Macherey-Nagel, Betlehem, PA, USA). Concentration and quality of the DNA was analyzed with an ND-1000 Spectrometer (NanoDrop Technologies, Wilmington, DE, USA) and gel electrophoresis. Genotyping of 90 RILs and parents was conducted using Illumina Golden Gate SNP assay and AFLP procedure, and co-dominant scoring of markers. Gene-based SNPs were assayed using mostly OPA5 (113 markers), with 28 remaining markers coming from combined OPA1, OPA2, and OPA3 (http://compgenomics.ucdavis.edu/compositae_SNP.php). The AFLP marker data were generated using 13 *Eco*RI/*Mse*I primer combinations to assay 179 anonymous loci. Seed color (*w* gene^43^) scored on RILs was included into the set of segregating markers. The GR × Ice linkage map was constructed using MapDisto 1.7.5 software^44^. The LOD threshold of 3 was used to group markers into linkage groups. The GR × Ice map was aligned to the ultrahigh-density reference genetic map^31^ to keep the numbering and orientation of linkage groups consistent. When two groups of markers were associated with a single chromosomal linkage group of the reference map, the two groups were labeled ‘a' and ‘b', respectively (e.g. LG 4a and LG 4b). Alignments of linkage maps was done with Mapchart 2.2^45^. Marker loci with distorted segregation of alleles were identified using MapDisto.

### Disease evaluation

Lettuce reaction to downy mildew was assessed in five field and two laboratory experiments. Field experiments were not artificially inoculated with *B. lactucae*, because natural infection occurs annually in tested locations. Four experiments (F-2008, F-2009, F-2012a, F-2012b) were conducted in Salinas, California between 2008 and 2012 and one experiment (F-2011) was conducted in The Netherlands in 2011 (Table 2). Experiments in Salinas were performed using a randomized complete block design with two or three replications. Plants were seeded in two rows 35 cm apart on 102 cm wide beds (center-to-center), 5 m per genotype, and thinned to the final spacing of ca. 30 cm between plants within a seedline, resulting in ca. 30 plants per replicate. Crop cultivation was done using standard cultural practices except that fungicide treatment was not applied. In addition, fall experiments were sprinkler irrigated for approximately 10 min. every other evening, to maintain high relative humidity favorable to development of downy mildew. The F-2011 experiment in The Netherlands utilized an alpha lattice design with two replications of 20 plants each. All experiments included 90 RILs, both parents, and a susceptible check (cultivar Salinas).

Experiments were inspected weekly and the first rating was performed about a week after disease symptoms were first observed on the susceptible check. The week of the first evaluation was numbered week 1 (WK1), regardless of the date. Subsequent disease evaluations were performed in weekly or biweekly intervals depending on disease progress. The last disease evaluation was always performed at harvest maturity. If disease developed late in the growing season (F-2008, and F-2012a), only a single evaluation at harvest maturity was performed. Disease was visually evaluated using a 0 to 5 rating scale that combined both disease incidence and symptom severity (Table 5). Half points were used to describe material that did not clearly fit into a single category, thus allowing for a more gradual increase in rating. In Salinas, an overall rating was given to the whole plot with ca. 30 plants. In The Netherlands, the mean score per plot was calculated from six individually evaluated plants. Disease scores were averaged across all blocks within experiments prior to data analyses. In experiments with two or more disease evaluations, multiple ratings were combined into a single value that reflected disease progress from WK1 until the current evaluation. This value is termed the area under the disease progress steps (AUDPS)^46^.

Two laboratory experiments (L-2012a, and L-2012b) were performed to evaluate disease resistance at the seedling stage. The L-2012a experiment used *B. lactucae* isolate C11O1352 that is virulent on all known *Dm* genes except *Dm17* and *Dm36*. The L-2012b experiment used isolate C11O1345 that is virulent on all known *Dm* genes except *Dm6*, *Dm17*, *Dm37*, and *Dm38*. To test for resistance, seedlings of both parents, RILs, and susceptible check were sprayed with *B. lactucae* conidia suspension (1 × 10^4^ conidia per ml) seven days after sowing. Inoculated seedlings were maintained in clear plastic boxes at 15°C and 14-h light period. Sporulation (emergence of conidiospores) was assessed on at least 15 seedlings per genotype at 7 and 12 DAI. Sporulation was visually evaluated and scored using a 0 to 3 scale, where 0 = no sporulation, 1 = sporulation on a few seedlings, 2 = sporulation on ~ 50% of seedlings, and 3 = sporulation on ≥ 75% of seedlings.

To determine the virulence phenotypes of *B. lactucae*^40^ that occurred naturally in field experiments, isolates were collected at plant harvest maturity. Leaves with sporulating lesions were harvested from both parents, the susceptible check, and randomly selected RILs that were evenly distributed throughout the field. The virulence phenotypes of *B. lactucae* isolates were determined in laboratory by inoculation onto seedlings of a differential set of cultivars that express all the known *Dm* genes^47^,^48^,^49^. Virulence was assessed as described for the laboratory experiments.

### Data analyses

Normality of distribution of resistance data was tested with D'Agostino-Pearson test implemented in the QGene v. 4.3.9 software^50^. QTL mapping on datasets with normal distribution was performed in QGene using the Simple Interval Mapping (SIM) feature. QTL mapping on datasets with non-normal distribution was performed twice, once with parametric SIM test in QGene and the second time with a non-parametric Kruscal-Wallis test implemented in MapQTL v. 6 (Kyazma, Wageningen, The Netherlands). Unmapped markers that were not assigned to any linkage group were analyzed with single-marker regression (SMR) approach. Significance threshold for the LOD scores were determined at genome-wide α_0.05_ through permutations with 1,000 iterations. The scan interval in QGene was set at 1 cM. The percentage of phenotypic variation (R^2^%) explained by QTLs and additive effects of alleles were calculated by QGene in the SIM mode. Similarly, 1-LOD support intervals^51^ of the QTLs locations were calculated from SIM output. Mixed-model composite interval mapping (MCIM) was applied to test for QTLs, interactions between significant QTLs, and QTL × environment (Q × E) interaction on final data from the seven experiments (Table 2). MCIM was performed using the default setting of QTLNetwork v. 2.1^37^. Threshold value to declare QTLs or interactions significant was P ≤ 0.001. In addition to analyses performed on data from individual experiments, SIM was conducted in QGene on an integrated rating that combined data from the five field and two laboratory experiments into a single value. The integrated rating was calculated using Bradley-Terry model for paired comparisons^52^ according to Simko and Pechenick^53^. Because integrated rating approach produces values on a latent scale, the values were converted into a 0 to 100 scale, where the overall most resistant RIL received the value of 0, and the least resistant RIL received the value of 100.

The potential for marker-assisted selection (MAS) in the GR × Ice population was evaluated for a combination of markers that were the most closely linked to the two significant QTLs (see results) detected in multiple experiments. Because *F_6_* RILs are highly homozygous, only four genotypes of Grand Rapids alleles (*g*) and Iceberg alleles (*i*) were predominant at the two markers (*gg*-*gg*, *gg*-*ii*, *ii*-*gg*, and *ii*-*ii*). Five RILs with heterozygous genotypes (*gi* or *ig*) at one or both markers were omitted from the analysis, because the small sample size did not allow for proper statistical analysis of these genotypes. The analysis of means (ANOM) was applied to determine which of the four allelic combinations leads to resistance that is significantly different from the overall resistance in the population.

The lettuce genome sequence (https://lgr.genomecenter.ucdavis.edu) and ultra-high-density molecular linkage map^31^ were used to investigate whether there were candidate resistance genes^11^,^12^,^54^ located in the chromosomal regions within the QTL intervals. Candidate resistance genes were identified as those having significant BLAST^55^ similarity (≤ 1 × *e*^−20^ threshold) to genes involved in resistance from other species^12^,^54^.

Statistical analyses of ANOM, Pearson and Spearman correlations were performed using JMP 6.0.3 software (SAS Institute, Cary, NC, USA).

## Supplementary Material

Supplementary Information Supplementary information

## Figures and Tables

**Table 1:** Description of linkage groups of the Grand Rapids × Iceberg linkage map

Linkage group	Number of AFLP markers	Number of SNP markers	Total number of markers	Length of linkage group in cM	Average interval between two subsequent markers in cM	Number of anchor markers^c^	Length of linkage group in reference map in cM^d^	Percentage covering of reference map^e^	Number of markers with distorted segregation
LG 1	31	3	34	71	2.1	2	144	49	0
LG 2^a^	17	29	46	168	3.7	16	152	111	2
LG 3	21	2	23	78	3.6	2	174	45	0
LG 4^a^	26	22	48	143	3.1	12	234	61	7
LG 5^a^	17	15	32	90	2.9	8	234	38	2
LG 6^a^	2	15	17	58	3.6	10	243	40	2
LG 7	20	17	38^b^	133	3.6	11	142	94	2
LG 8^a^	20	19	39	178	4.7	12	204	87	1
LG 9	22	9	31	98	3.3	7	159	62	10
Total	176	131	308^b^	1,017	3.3	80	1,586	64	26

^a^Combined data for two sections of this linkage group.

^b^Data include a single phenotypic marker for white seed color (*w* gene).

^c^SNP markers with known position on both Grand Rapids × Iceberg linkage map and reference map.

^d^Reference map was based on segregation of markers in the interspecific (*L. sativa* × *L. serriola*) mapping population^31^.

^e^Length of Grand Rapids × Iceberg linkage map expressed as a percentage of length of reference linkage map.

**Table 2:** Description of experiments evaluating lettuce reaction to downy mildew

						Scores at last evaluation				
Experiment^a^	Location	Evaluation period	No. of replications	Evaluation weeks^b^	Rating scale	RILs Mean	RILs Range	Salinas^d^	Grand Rapids	Iceberg
F-2008	Salinas, CA	Fall	2	1	0 – 5	1.3	0–3.8	4.6	0.6	0.6
F-2009	Salinas, CA	Fall	3	1, 3, 5	0 – 5	2.4	1–4	4.9	2	1
F-2011	Netherlands	Fall	2	1, 2, 5	0 – 5	2.1	0.2–4	4.7	2	1
F-2012a	Salinas, CA	Summer	3	1	0 – 5	0.4	0–1.7	3.3	0.8	0.4
F-2012b	Salinas, CA	Fall	3	1, 2, 3	0 – 5	1.4	0–3	4.5	1.8	1.5
L-2012a	Laboratory	n.a.	2	1^c^	0 – 3	0.2	0–1	1	0	0
L-2012b	Laboratory	n.a.	2	1^c^	0 – 3	0.3	0–1	1	1	0

^a^F and L indicate field or laboratory conditions, the number shows years when the experiments were conducted.

^b^Evaluations in weeks after downy mildew symptoms were first observed on susceptible control. Last evaluations at field experiments were always performed at harvest maturity. If disease appeared late in season, only a single evaluation at harvest maturity was performed.

^c^Two evaluations of laboratory tests were conducted, but only the first evaluation at one week after inoculation was used for QTL mapping (see results for detailed explanation).

^d^Cultivar Salinas was susceptible control.

**Table 3:** QTLs detected for resistance to downy mildew in the Grand Rapids × Iceberg population^a^

QTL	Experiment	Disease assessment	Week of assessment	LOD^c^	R^2^%^d^	*gg* genotype^e^ Mean ± S.E.	*ii* genotype^f^ Mean ± S.E.
*qDM2.1*	F-2009	Rating	3	4.8	22	2.60 ± 0.13	1.64 ± 0.15
		Rating	5	3.2	15	2.65 ± 0.12	2.06 ± 0.09
		AUDPS^b^	3	4.1	19	68.33 ± 3.16	48.48 ± 3.19
		AUDPS	5	4.5	20	86.91 ± 3.67	62.90 ± 3.65
	F-2011	Rating	2	3.5	16	1.96 ± 0.07	1.53 ± 0.07
		AUDPS	2	3.0	14	20.63 ± 0.95	15.86 ± 0.72
		AUDPS	5	2.8	13	97.85 ± 4.53	75.28 ± 4.18
	F-2012b	AUDPS	3	3.0	12	21.52 ± 0.87	16.81 ± 0.81
	L-2012a	Sporulation	1	3.2	15	0.31 ± 0.07	0.02 ± 0.03
	Integrated	0 – 100	n.a.	4.5	21	56.85 ± 3.48	33.84 ± 2.83
*qDM5.1*	F-2008	Rating	1	2.8	13	1.69 ± 0.15	0.90 ± 0.12
	F-2011	Rating	1	3.7	17	1.08 ± 0.06	0.68 ± 0.06
		Rating	2	4.4	20	2.01 ± 0.07	1.52 ± 0.07
		Rating	5	3.1	14	2.50 ± 0.13	1.81 ± 0.13
		AUDPS	2	4.6	21	21.64 ± 0.83	15.43 ± 0.83
		AUDPS	5	3.6	17	102.16 ± 4.14	73.77 ± 4.40
	L-2012b	Sporulation	1	2.9	14	0.51 ± 0.07	0.14 ± 0.05
	Integrated	0 – 100	n.a	3.4	16	56.82 ± 3.44	38.41 ± 3.31

^a^No significant QTLs were detected from F-2009 rating in week 1, F-2012a rating, and F-2012b ratings in weeks 1, 2, 3 and AUDPS in week 2.

^b^AUDPS - the area under the disease progress steps is calculated from individual ratings and combines disease progress from the first evaluation until the current evaluation^46^. This value is always larger than sum of individual ratings.

^c^LOD – Logarithm of the odds score for QTLs calculated by simple interval mapping.

^d^R^2^% - Percentage of the total phenotypic variation of the trait explained by the QTL.

^e^Mean value and standard error for RILs homozygous for Grand Rapids allele at the QTL.

^f^Mean value and standard error for RILs homozygous for Iceberg allele at the QTL.

**Table 4:** QTLs detected in the Grand Rapids × Iceberg population by mixed-model based composite interval mapping performed on final ratings of the seven experiments

QTL	LG	Position in cM	Range in cM^a^	a^b^ ± S.E.	Significance^c^
*qDM2.1*	2	63.6	62.6 – 63.7	0.117 ± 0.022	> 6.0
*qDM5.1*	5	29.6	28.6 – 29.8	0.147 ± 0.022	> 6.0
*qDM9.1*	9	30.1	29.1 – 31.1	−0.088 ± 0.024	3.5

^a^Range of the QTL support interval calculated by QTLNetwork software.

^b^Additive main effect (± standard error) of the QTL. Positive values indicate that alleles for elevated resistance originate from cv. Iceberg, while negative values indicate that alleles for elevated resistance originate from cv. Grand Rapids.

^c^Significance of the QTL expressed as negative common logarithm of P value.

**Table 5:** Visual rating scale for evaluating reaction of lettuce to downy mildew infection in field experiments

Rating^a^	Description
0	No lesions, all plants disease free.
1	Sporadic lesions, with less than one lesion per plant on average. Limited pathogen sporulation.
2	Up to two lesions per plant. Lesions limited to outer/lower leaves.
3	Three to ten lesions per plant. Lesions on outer/lower leaves. Disease spreading to wrapper/upper leaves.
4	More than ten lesions per plant. Many lesions on outer/lower leaves. Moderate number of lesions on wrapper/upper leaves.
5	Severe plant infection. Merging of individual lesions into areas covering large parts of the plant leaves. Heavy pathogen sporulation and/or large number of necrotic leaf areas.

^a^Half points were given to material that did not fit into a single category.
