# Supplementary material for: Identification of QTLs conferring resistance to downy mildew in legacy cultivars of lettuce
Source: Sci Rep. 2013 Oct 7;3:2875. doi: 10.1038/srep02875 (PMC3791445; doi:10.1038/srep02875)

Supplementary Information

Identification of QTLs conferring resistance to downy mildew in legacy cultivars of lettuce

Ivan Simko[1*], Amy J. Atallah[1], Oswaldo E. Ochoa[2], Rudie Antonise[3], Carlos H. Galeano[1,2], Maria-Jose Truco[2], and Richard W. Michelmore[2]
[1] U.S. Department of Agriculture, Agricultural Research Service, U.S. Agricultural Research Station, 1636 E. Alisal St, Salinas, CA 93905, USA
[2] The Genome Center and Department of Plant Sciences, University of California, Davis, CA 95616, USA
[3] Keygene N.V., P.O. Box 216 6700 AE Wageningen, The Netherlands
[*] Corresponding author

Supplementary Table

Table S1: **Correlation coefficients of traits means.** Pearson's coefficient is above black diagonal; Spearman's coefficient is below diagonal. Significance level is indicated by darker green color.

Experiment	Week	Evaluation	F-2008 WK1 Rating	F-2009 WK1 Rating	F-2009 WK3 Rating	F-2009 WK3 AUDPS	F-2009 WK5 Rating	F-2009 WK5 AUDPS	F-2011 WK1 Rating	F-2011 WK2 Rating	F-2011 WK2 AUDPS	F-2011 WK5 Rating	F-2011 WK5 AUDPS	F-2012a WK1 Rating	F-2012b WK1 Rating	F-2012b WK2 Rating	F-2012b WK2 AUDPS	F-2012b WK3 Rating	F-2012b WK3 AUDPS	L-2012a WK1 Sporulation	L-2012b WK1 Sporulation	Integrated n.a. 0-100
F-2008	WK1	Rating		0.36	0.35	0.41	0.42	0.43	0.50	0.54	0.56	0.45	0.52	0.53	0.02	0.31	0.28	0.44	0.42	0.23	-0.05	0.67
F-2009	WK1	Rating	0.42		0.48	0.82	0.44	0.78	0.31	0.30	0.33	0.28	0.32	0.28	-0.07	0.21	0.16	0.32	0.28	0.28	0.10	0.47
F-2009	WK3	Rating	0.36	0.49		0.88	0.65	0.89	0.39	0.45	0.45	0.32	0.39	0.21	-0.05	0.21	0.17	0.25	0.24	0.27	0.11	0.38
F-2009	WK3	AUDPS	0.42	0.83	0.89		0.65	0.99	0.41	0.45	0.46	0.35	0.42	0.28	-0.07	0.24	0.19	0.33	0.30	0.32	0.12	0.45
F-2009	WK5	Rating	0.39	0.43	0.64	0.64		0.74	0.39	0.47	0.46	0.41	0.46	0.41	0.01	0.28	0.25	0.36	0.35	0.25	0.08	0.42
F-2009	WK5	AUDPS	0.44	0.80	0.90	0.99	0.76		0.43	0.48	0.49	0.38	0.45	0.32	-0.06	0.26	0.21	0.35	0.33	0.33	0.12	0.46
F-2011	WK1	Rating	0.47	0.32	0.40	0.41	0.32	0.41		0.73	0.85	0.61	0.67	0.47	0.04	0.40	0.36	0.35	0.41	0.26	0.09	0.55
F-2011	WK2	Rating	0.52	0.31	0.46	0.44	0.39	0.46	0.66		0.94	0.84	0.89	0.43	0.02	0.37	0.32	0.44	0.44	0.38	0.22	0.65
F-2011	WK2	AUDPS	0.54	0.34	0.47	0.46	0.39	0.47	0.91	0.94		0.79	0.92	0.48	0.03	0.41	0.37	0.43	0.46	0.35	0.18	0.65
F-2011	WK5	Rating	0.45	0.31	0.35	0.37	0.39	0.38	0.57	0.84	0.80		0.98	0.38	0.09	0.32	0.31	0.39	0.41	0.43	0.22	0.65
F-2011	WK5	AUDPS	0.49	0.32	0.36	0.38	0.37	0.40	0.76	0.93	0.88	0.97		0.44	0.07	0.37	0.35	0.43	0.45	0.42	0.21	0.67
F-2012a	WK1	Rating	0.55	0.30	0.21	0.26	0.39	0.29	0.45	0.41	0.45	0.40	0.44		0.12	0.44	0.42	0.57	0.58	0.27	0.07	0.75
F-2012b	WK1	Rating	0.03	-0.03	-0.05	-0.06	0.06	-0.04	0.14	0.09	0.12	0.12	0.11	0.16		0.23	0.55	0.15	0.38	0.01	0.32	0.18
F-2012b	WK2	Rating	0.36	0.23	0.19	0.19	0.25	0.21	0.43	0.38	0.42	0.35	0.37	0.50	0.31		0.95	0.52	0.82	0.18	0.15	0.56
F-2012b	WK2	AUDPS	0.31	0.18	0.13	0.13	0.22	0.16	0.41	0.35	0.40	0.35	0.36	0.49	0.52	0.95		0.50	0.86	0.16	0.24	0.55
F-2012b	WK3	Rating	0.44	0.41	0.26	0.33	0.29	0.34	0.33	0.36	0.36	0.35	0.35	0.55	0.14	0.52	0.51		0.88	0.38	0.08	0.83
F-2012b	WK3	AUDPS	0.45	0.33	0.25	0.27	0.32	0.30	0.43	0.42	0.44	0.41	0.42	0.61	0.38	0.84	0.84	0.88		0.31	0.18	0.81
L-2012a	WK1	Sporulation	0.22	0.34	0.30	0.36	0.22	0.36	0.28	0.37	0.37	0.40	0.40	0.21	0.05	0.16	0.15	0.36	0.31		0.18	0.53
L-2012b	WK1	Sporulation	-0.01	0.09	0.09	0.10	0.08	0.10	0.15	0.26	0.26	0.21	0.22	0.10	0.28	0.15	0.22	0.09	0.18	0.19		0.33
Integrated	n.a.	0-100	0.66	0.40	0.37	0.44	0.47	0.48	0.55	0.68	0.67	0.67	0.70	0.72	0.21	0.55	0.55	0.83	0.80	0.54	0.31	

P E A R S O N

S P E A R M A N

Significant at *P*-value
< 0.05
< 0.01
< 0.001

Supplementary Information

Identification of QTLs conferring resistance to downy mildew in legacy cultivars of lettuce

Ivan Simko[1*], Amy J. Atallah[1], Oswaldo E. Ochoa[2], Rudie Antonise[3], Carlos H. Galeano[1,2], Maria-Jose Truco[2], and Richard W. Michelmore[2]
[1] U.S. Department of Agriculture, Agricultural Research Service, U.S. Agricultural Research Station, 1636 E. Alisal St, Salinas, CA 93905, USA
[2] The Genome Center and Department of Plant Sciences, University of California, Davis, CA 95616, USA
[3] Keygene N.V., P.O. Box 216 6700 AE Wageningen, The Netherlands
[*] Corresponding author

Supplementary Figure

Figure S2: **Avirulence genes detected in *B. lactucae* isolates.** Fifty-eight samples of the pathogen were collected in Salinas during 2008 - 2012 period. The avirulence genes that were never detected are *Avr5/8*, *Avr7*, *Avr11*, *Avr12*, *Avr13*, *Avr14*, *Avr15*, *Avr18*, *Avr18/32*, and *Avr40*.

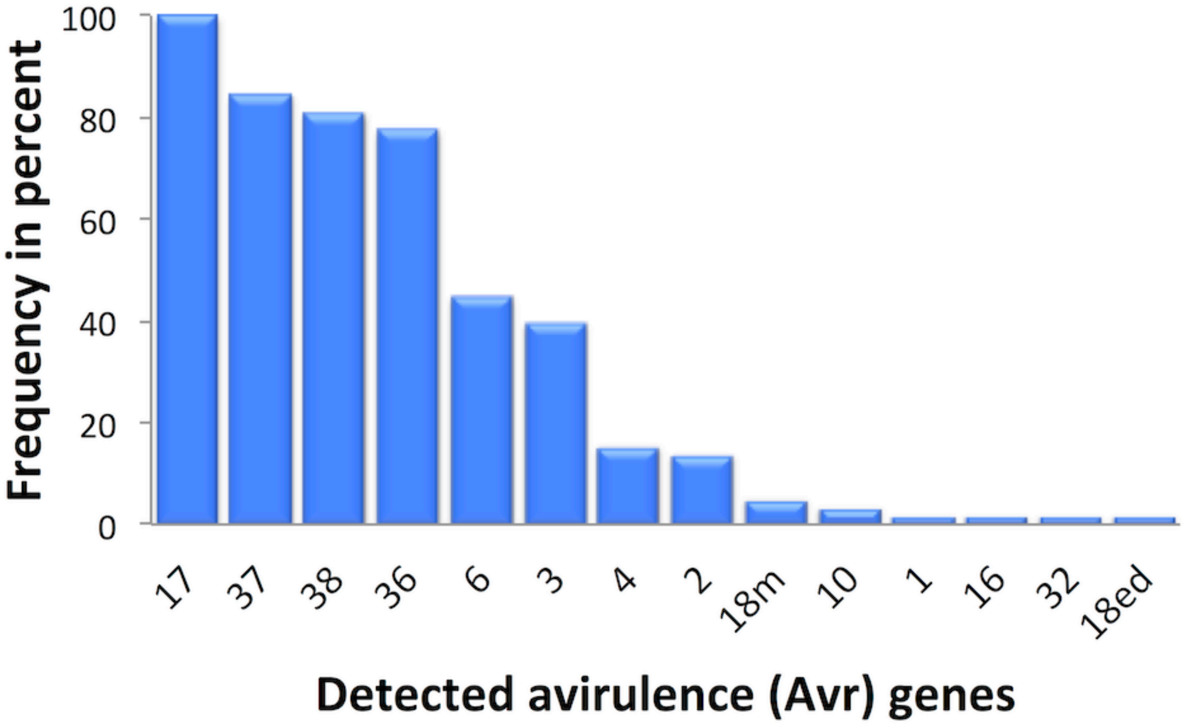

Supplement: Supplementary Information [file srep02875-s1.pdf]
